# Dichlorido{2-[2-(dimethyl­ammonio)ethyl­imino­meth­yl]-6-methoxy­phenolato}zinc(II)

**DOI:** 10.1107/S1600536809028220

**Published:** 2009-07-22

**Authors:** Amitabha Datta, Nien-Tsu Chuang, Jui-Hsien Huang, Hon Man Lee

**Affiliations:** a National Changhua University of Education, Department of Chemistry, Changhua, Taiwan 50058

## Abstract

The structure of the title complex, [ZnCl_2_(C_12_H_18_N_2_O_2_)], contains a zwitterionic Schiff base ligand. The complex adopts a distorted tetra­hedral coordination geometry around the metal centre with the Schiff base ligand coordinated in a bidentate fashion *via* the imine N and phenolate O atoms. In the crystal, inter­molecular N—H⋯O and C—H⋯Cl hydrogen bonds link the mol­ecules into chains parallel to the *c*-glide planes.

## Related literature

For bidentate Schiff base–Zn(II) complexes, see: Qiu (2006 ▶); Wang & Qiu (2006 ▶); Ye & You (2008 ▶); You (2005*a* ▶,*b* ▶); Zhu (2008 ▶).
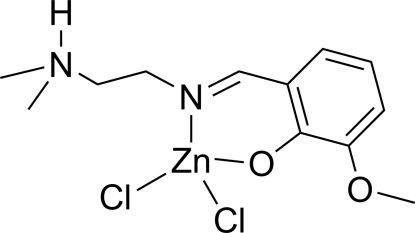


## Experimental

### 

#### Crystal data

[ZnCl_2_(C_12_H_18_N_2_O_2_)]
*M* *_r_* = 358.55
Monoclinic, 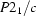

*a* = 8.5621 (6) Å
*b* = 16.9642 (12) Å
*c* = 13.1106 (7) Å
β = 127.732 (3)°
*V* = 1506.08 (17) Å^3^
*Z* = 4
Mo *K*α radiation
μ = 1.98 mm^−1^
*T* = 298 K
0.32 × 0.25 × 0.22 mm

#### Data collection

Bruker SMART APEXII diffractometer
Absorption correction: multi-scan (*SADABS*; Sheldrick, 2003 ▶) *T*_min_ = 0.552, *T*_max_ = 0.649
8355 measured reflections
2960 independent reflections
2439 reflections with *I* > 2σ(*I*)
*R*_int_ = 0.030

#### Refinement

*R*[*F* ^2^ > 2σ(*F* ^2^)] = 0.028
*wR*(*F* ^2^) = 0.075
*S* = 1.01
2960 reflections
175 parameters
H-atom parameters constrained
Δρ_max_ = 0.35 e Å^−3^
Δρ_min_ = −0.34 e Å^−3^

### 

Data collection: *APEX2* (Bruker, 2004 ▶); cell refinement: *SAINT* (Bruker, 2004 ▶); data reduction: *SAINT*; program(s) used to solve structure: *SHELXTL* (Sheldrick, 2008 ▶); program(s) used to refine structure: *SHELXTL*; molecular graphics: *SHELXTL*; software used to prepare material for publication: *SHELXTL*.

## Supplementary Material

Crystal structure: contains datablocks I, global. DOI: 10.1107/S1600536809028220/bh2237sup1.cif

Structure factors: contains datablocks I. DOI: 10.1107/S1600536809028220/bh2237Isup2.hkl

Additional supplementary materials:  crystallographic information; 3D view; checkCIF report

## Figures and Tables

**Table 1:** Hydrogen-bond geometry (Å, °)

*D*—H⋯*A*	*D*—H	H⋯*A*	*D*⋯*A*	*D*—H⋯*A*
N1—H1⋯O1^i^	0.91	1.97	2.842 (2)	161
N1—H1⋯O2^i^	0.91	2.38	2.985 (2)	124
C5—H5⋯Cl2^i^	0.93	2.80	3.617 (2)	148

Symmetry code: (i) 
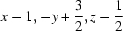
.

**Fig. 1.**
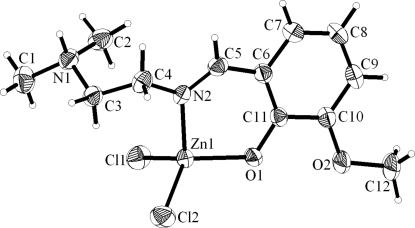
The structure of the title complex, showing 50% displacement ellipsoids for non-H atoms. The H atoms are depicted by circles of arbitrary radius.

**Fig. 2.**
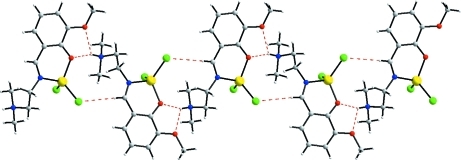
A packing diagram of the title compound showing the intermolecular hydrogen bonds (dashed lines).

## supplementary crystallographic information

### Comment

The Schiff base 2-((2-(dimethylamino)ethylimino)methyl)-6-methoxyphenol reacts with zinc(II) chloride in methanol to form the title complex. It adopts tetrahedron coordination geometry with the Schiff base ligand coordinated *via* the imine N and the phenolate O atoms. A formal proton transfer of the phenolic hydrogen to the amine N atom occurred leading to the formation of the neutral O/N-bidentate ligand bearing a pendant ammonium group.

Bidentate Schiff base Zn(II) complexes similar to the title complex were reported in the literature (Qiu, 2006; Wang & Qiu, 2006; Ye & You, 2008; You, 2005*a*, *b*; Zhu, 2008).

Classical intermolecular H-bonds of the type N—H···O and non-classical H-bonds of the type C—H···Cl exist (Table 1). These H-bonds link the complex into one-dimensional hydrogen bonded chains.

### Experimental

To an aqueous solution (20 ml) of zinc(II) chloride (0.136 g, 1.00 mmol), a methanolic solution of the Schiff base 2-((2-(dimethylamino)ethylimino)methyl)-6-methoxyphenol (1.00 mmol) was added. The resulting mixture was kept at room temperature yielding brown block-like crystals suitable for X-ray diffraction after few days. Crystals were isolated by filtration and were dried in the air.

### Refinement

All the H atoms were positioned geometrically and refined as riding atoms, with C_aryl_—H = 0.93, C_methyl_—H = 0.96, C_methylene_—H = 0.97, and N—H = 0.91 Å, while *U*_iso_(H) = 1.5*U*_eq_(C) for the methyl H atoms and *U*_iso_(H) = 1.2*U*_eq_(C) for all the other H atoms.

### Crystal data

**Table:**

[ZnCl_2_(C_12_H_18_N_2_O_2_)]	*F*(000) = 736
*M**_r_* = 358.55	*D*_x_ = 1.581 Mg m^−^^3^
Monoclinic, *P*2_1_/*c*	Mo *K*α radiation, λ = 0.71073 Å
Hall symbol: -P 2ybc	Cell parameters from 4320 reflections
*a* = 8.5621 (6) Å	θ = 2.4–26.0°
*b* = 16.9642 (12) Å	µ = 1.98 mm^−^^1^
*c* = 13.1106 (7) Å	*T* = 298 K
β = 127.732 (3)°	Block, brown
*V* = 1506.08 (17) Å^3^	0.32 × 0.25 × 0.22 mm
*Z* = 4	

### Data collection

**Table:**

Bruker SMART APEXII diffractometer	2960 independent reflections
Radiation source: fine-focus sealed tube	2439 reflections with *I* > 2σ(*I*)
graphite	*R*_int_ = 0.030
ω scans	θ_max_ = 26.0°, θ_min_ = 2.3°
Absorption correction: multi-scan (*SADABS*; Sheldrick, 2003)	*h* = −10→9
*T*_min_ = 0.552, *T*_max_ = 0.649	*k* = −20→20
8355 measured reflections	*l* = −10→16

### Refinement

**Table:**

Refinement on *F*^2^	Primary atom site location: structure-invariant direct methods
Least-squares matrix: full	Secondary atom site location: difference Fourier map
*R*[*F*^2^ > 2σ(*F*^2^)] = 0.028	Hydrogen site location: inferred from neighbouring sites
*wR*(*F*^2^) = 0.075	H-atom parameters constrained
*S* = 1.01	*w* = 1/[σ^2^(*F*_o_^2^) + (0.0461*P*)^2^] where *P* = (*F*_o_^2^ + 2*F*_c_^2^)/3
2960 reflections	(Δ/σ)_max_ = 0.001
175 parameters	Δρ_max_ = 0.35 e Å^−^^3^
0 restraints	Δρ_min_ = −0.34 e Å^−^^3^
0 constraints	

### Fractional atomic coordinates and isotropic or equivalent isotropic displacement parameters (Å^2^)

**Table:**

	*x*	*y*	*z*	*U*_iso_*/*U*_eq_	
Zn1	0.68123 (3)	0.747503 (13)	0.52685 (2)	0.02988 (10)	
Cl1	0.60094 (9)	0.72171 (4)	0.65804 (6)	0.04492 (16)	
Cl2	0.86631 (8)	0.65127 (4)	0.53283 (6)	0.04647 (17)	
O1	0.7870 (2)	0.85437 (8)	0.55417 (13)	0.0336 (3)	
O2	0.9469 (2)	0.99419 (9)	0.62283 (15)	0.0432 (4)	
N1	0.1190 (3)	0.63462 (11)	0.31873 (17)	0.0357 (4)	
H1	0.0110	0.6259	0.2358	0.043*	
N2	0.4369 (2)	0.76199 (10)	0.34509 (17)	0.0285 (4)	
C1	0.1242 (4)	0.57202 (15)	0.4010 (2)	0.0512 (6)	
H1A	0.0034	0.5732	0.3910	0.077*	
H1B	0.2336	0.5813	0.4897	0.077*	
H1C	0.1388	0.5214	0.3751	0.077*	
C2	0.0953 (3)	0.71360 (15)	0.3580 (2)	0.0442 (6)	
H2A	0.0800	0.7530	0.2999	0.066*	
H2B	0.2101	0.7253	0.4441	0.066*	
H2C	−0.0194	0.7133	0.3552	0.066*	
C3	0.2983 (3)	0.62794 (13)	0.3237 (2)	0.0364 (5)	
H3A	0.2912	0.5786	0.2837	0.044*	
H3B	0.4144	0.6258	0.4134	0.044*	
C4	0.3242 (3)	0.69479 (13)	0.2580 (2)	0.0354 (5)	
H4A	0.3921	0.6749	0.2253	0.043*	
H4B	0.1951	0.7132	0.1850	0.043*	
C5	0.3886 (3)	0.83097 (13)	0.2944 (2)	0.0324 (5)	
H5	0.2768	0.8338	0.2087	0.039*	
C6	0.4879 (3)	0.90494 (12)	0.35490 (19)	0.0302 (5)	
C7	0.3821 (3)	0.97261 (14)	0.2809 (2)	0.0393 (5)	
H7	0.2582	0.9666	0.2018	0.047*	
C8	0.4579 (4)	1.04639 (14)	0.3232 (2)	0.0432 (6)	
H8	0.3841	1.0903	0.2750	0.052*	
C9	0.6463 (3)	1.05555 (13)	0.4387 (2)	0.0397 (5)	
H9	0.6988	1.1059	0.4674	0.048*	
C10	0.7563 (3)	0.99069 (12)	0.5115 (2)	0.0321 (5)	
C11	0.6771 (3)	0.91289 (12)	0.47493 (19)	0.0280 (4)	
C12	1.0452 (4)	1.06907 (14)	0.6573 (3)	0.0537 (7)	
H12A	0.9871	1.1044	0.6828	0.081*	
H12B	1.0314	1.0907	0.5845	0.081*	
H12C	1.1827	1.0622	0.7276	0.081*	

### Atomic displacement parameters (Å^2^)

**Table:**

	*U*^11^	*U*^22^	*U*^33^	*U*^12^	*U*^13^	*U*^23^
Zn1	0.02911 (15)	0.02351 (15)	0.03349 (16)	−0.00104 (9)	0.01735 (12)	0.00050 (9)
Cl1	0.0429 (3)	0.0566 (4)	0.0423 (3)	0.0001 (3)	0.0297 (3)	0.0034 (3)
Cl2	0.0428 (3)	0.0418 (3)	0.0553 (4)	0.0101 (3)	0.0303 (3)	−0.0005 (3)
O1	0.0304 (7)	0.0224 (8)	0.0340 (8)	−0.0004 (6)	0.0126 (6)	0.0016 (6)
O2	0.0423 (9)	0.0256 (8)	0.0426 (9)	−0.0090 (7)	0.0163 (8)	−0.0024 (7)
N1	0.0308 (9)	0.0373 (11)	0.0342 (10)	−0.0060 (8)	0.0175 (8)	−0.0036 (8)
N2	0.0267 (8)	0.0282 (10)	0.0314 (9)	−0.0047 (7)	0.0183 (8)	−0.0039 (7)
C1	0.0598 (16)	0.0482 (16)	0.0506 (15)	−0.0093 (12)	0.0363 (14)	0.0020 (12)
C2	0.0353 (12)	0.0465 (15)	0.0497 (14)	0.0028 (11)	0.0254 (11)	−0.0053 (11)
C3	0.0323 (11)	0.0282 (12)	0.0454 (13)	−0.0019 (9)	0.0220 (10)	−0.0063 (9)
C4	0.0360 (11)	0.0366 (13)	0.0353 (12)	−0.0084 (9)	0.0227 (10)	−0.0113 (9)
C5	0.0286 (10)	0.0379 (13)	0.0312 (11)	0.0001 (9)	0.0186 (9)	0.0028 (9)
C6	0.0325 (10)	0.0273 (11)	0.0343 (11)	0.0011 (8)	0.0222 (9)	0.0028 (8)
C7	0.0364 (12)	0.0376 (13)	0.0380 (12)	0.0038 (10)	0.0197 (10)	0.0080 (10)
C8	0.0462 (14)	0.0292 (13)	0.0490 (14)	0.0094 (10)	0.0264 (12)	0.0145 (10)
C9	0.0508 (14)	0.0250 (12)	0.0480 (14)	−0.0013 (10)	0.0326 (12)	0.0041 (10)
C10	0.0364 (12)	0.0264 (11)	0.0334 (11)	−0.0013 (9)	0.0214 (10)	0.0004 (9)
C11	0.0317 (10)	0.0260 (11)	0.0315 (11)	0.0007 (8)	0.0219 (9)	0.0027 (8)
C12	0.0561 (16)	0.0275 (14)	0.0609 (17)	−0.0159 (11)	0.0272 (14)	−0.0082 (11)

### Geometric parameters (Å, °)

**Table:**

Zn1—O1	1.9590 (14)	C3—C4	1.521 (3)
Zn1—N2	2.0034 (18)	C3—H3A	0.9700
Zn1—Cl2	2.2429 (6)	C3—H3B	0.9700
Zn1—Cl1	2.2516 (6)	C4—H4A	0.9700
O1—C11	1.324 (2)	C4—H4B	0.9700
O2—C10	1.372 (3)	C5—C6	1.450 (3)
O2—C12	1.435 (3)	C5—H5	0.9300
N1—C2	1.493 (3)	C6—C11	1.414 (3)
N1—C1	1.495 (3)	C6—C7	1.415 (3)
N1—C3	1.500 (3)	C7—C8	1.362 (3)
N1—H1	0.9100	C7—H7	0.9300
N2—C5	1.283 (3)	C8—C9	1.389 (3)
N2—C4	1.479 (3)	C8—H8	0.9300
C1—H1A	0.9600	C9—C10	1.381 (3)
C1—H1B	0.9600	C9—H9	0.9300
C1—H1C	0.9600	C10—C11	1.426 (3)
C2—H2A	0.9600	C12—H12A	0.9600
C2—H2B	0.9600	C12—H12B	0.9600
C2—H2C	0.9600	C12—H12C	0.9600
			
O1—Zn1—N2	97.67 (6)	H3A—C3—H3B	107.5
O1—Zn1—Cl2	115.44 (5)	N2—C4—C3	112.94 (18)
N2—Zn1—Cl2	109.39 (5)	N2—C4—H4A	109.0
O1—Zn1—Cl1	111.30 (5)	C3—C4—H4A	109.0
N2—Zn1—Cl1	110.35 (5)	N2—C4—H4B	109.0
Cl2—Zn1—Cl1	111.80 (3)	C3—C4—H4B	109.0
C11—O1—Zn1	121.55 (13)	H4A—C4—H4B	107.8
C10—O2—C12	117.37 (18)	N2—C5—C6	127.7 (2)
C2—N1—C1	109.82 (19)	N2—C5—H5	116.2
C2—N1—C3	113.83 (16)	C6—C5—H5	116.2
C1—N1—C3	109.71 (18)	C11—C6—C7	120.1 (2)
C2—N1—H1	107.8	C11—C6—C5	125.47 (19)
C1—N1—H1	107.8	C7—C6—C5	114.34 (19)
C3—N1—H1	107.8	C8—C7—C6	121.4 (2)
C5—N2—C4	116.87 (19)	C8—C7—H7	119.3
C5—N2—Zn1	119.94 (14)	C6—C7—H7	119.3
C4—N2—Zn1	122.48 (14)	C7—C8—C9	119.5 (2)
N1—C1—H1A	109.5	C7—C8—H8	120.2
N1—C1—H1B	109.5	C9—C8—H8	120.2
H1A—C1—H1B	109.5	C10—C9—C8	120.7 (2)
N1—C1—H1C	109.5	C10—C9—H9	119.7
H1A—C1—H1C	109.5	C8—C9—H9	119.7
H1B—C1—H1C	109.5	O2—C10—C9	124.4 (2)
N1—C2—H2A	109.5	O2—C10—C11	114.14 (18)
N1—C2—H2B	109.5	C9—C10—C11	121.5 (2)
H2A—C2—H2B	109.5	O1—C11—C6	125.57 (19)
N1—C2—H2C	109.5	O1—C11—C10	117.83 (18)
H2A—C2—H2C	109.5	C6—C11—C10	116.59 (18)
H2B—C2—H2C	109.5	O2—C12—H12A	109.5
N1—C3—C4	114.78 (18)	O2—C12—H12B	109.5
N1—C3—H3A	108.6	H12A—C12—H12B	109.5
C4—C3—H3A	108.6	O2—C12—H12C	109.5
N1—C3—H3B	108.6	H12A—C12—H12C	109.5
C4—C3—H3B	108.6	H12B—C12—H12C	109.5
			
N2—Zn1—O1—C11	14.33 (16)	C11—C6—C7—C8	−0.5 (3)
Cl2—Zn1—O1—C11	130.12 (14)	C5—C6—C7—C8	−178.3 (2)
Cl1—Zn1—O1—C11	−101.07 (15)	C6—C7—C8—C9	2.7 (4)
O1—Zn1—N2—C5	−8.41 (17)	C7—C8—C9—C10	−0.6 (4)
Cl2—Zn1—N2—C5	−128.86 (16)	C12—O2—C10—C9	−7.6 (3)
Cl1—Zn1—N2—C5	107.74 (16)	C12—O2—C10—C11	172.5 (2)
O1—Zn1—N2—C4	161.59 (15)	C8—C9—C10—O2	176.4 (2)
Cl2—Zn1—N2—C4	41.14 (16)	C8—C9—C10—C11	−3.7 (3)
Cl1—Zn1—N2—C4	−82.26 (15)	Zn1—O1—C11—C6	−9.9 (3)
C2—N1—C3—C4	−48.9 (2)	Zn1—O1—C11—C10	171.41 (14)
C1—N1—C3—C4	−172.44 (19)	C7—C6—C11—O1	177.8 (2)
C5—N2—C4—C3	−146.55 (19)	C5—C6—C11—O1	−4.7 (3)
Zn1—N2—C4—C3	43.2 (2)	C7—C6—C11—C10	−3.5 (3)
N1—C3—C4—N2	88.5 (2)	C5—C6—C11—C10	174.0 (2)
C4—N2—C5—C6	−173.07 (19)	O2—C10—C11—O1	4.4 (3)
Zn1—N2—C5—C6	−2.5 (3)	C9—C10—C11—O1	−175.5 (2)
N2—C5—C6—C11	12.0 (4)	O2—C10—C11—C6	−174.43 (18)
N2—C5—C6—C7	−170.4 (2)	C9—C10—C11—C6	5.6 (3)

### Hydrogen-bond geometry (Å, °)

**Table:**

*D*—H···*A*	*D*—H	H···*A*	*D*···*A*	*D*—H···*A*
N1—H1···O1^i^	0.91	1.97	2.842 (2)	161
N1—H1···O2^i^	0.91	2.38	2.985 (2)	124
C5—H5···Cl2^i^	0.93	2.80	3.617 (2)	148

Symmetry codes: (i) *x*−1, −*y*+3/2, *z*−1/2.
